# Developing Bioengineered 3D-Printed Composite Scaffolds with Antimicrobial Potential for Bone Tissue Regeneration

**DOI:** 10.3390/jfb16060227

**Published:** 2025-06-19

**Authors:** Andreea Trifan, Eduard Liciu, Cristina Busuioc, Izabela-Cristina Stancu, Adela Banciu, Carmen Nicolae, Mihai Dragomir, Doru-Daniel Cristea, Rosina-Elena Sabău, David-Andrei Nițulescu, Alexandru Paraschiv

**Affiliations:** 1 3D Printing Laboratory, Center of Innovation and e-Health, “Carol Davila” University of Medicine and Pharmacy, 020021 Bucharest, Romania; andreea.trifan@umfcd.ro (A.T.);; 2 Faculty of Chemical Engineering and Biotechnologies, University Politehnica of Bucharest, 1-7 Gh. Polizu Street, 011061 Bucharest, Romania; 3 Faculty of Medical Engineering, University Politehnica of Bucharest, 1-7 Gh. Polizu Street, 011061 Bucharest, Romania; 4 REOROM Laboratory, Hydraulics Department, Politehnica University of Bucharest, 313 Splaiul Independenţei, 060042 Bucharest, Romania; 5 Special Components for Gas Turbines Department, Romanian Research and Development Institute for Gas Turbine COMOTI, 220D Iuliu Maniu, 061126 Bucharest, Romania

**Keywords:** bioglass, scaffolds, 3D printing, tissue engineering, hydrogels, biopolymers, composite materials

## Abstract

This research activity proposes to produce composite hydrogel–bioactive glass. The primary purpose of this research is to develop and optimize 3D-printed scaffolds using doped bioglass, aimed at enhancing bone regeneration in bone defects. The bioglass, a bioactive material known for its bone-bonding ability (SiO_2_–P_2_O_5_–CaO–Na_2_O), co-doped with europium and silver was synthesized and doped to improve its biological properties. This doped bioglass was then combined with a biocompatible hydrogel, chosen for its adequate cellular response and printability. The composite material was printed to form a scaffold, providing a structure that not only supports the damaged bone but also encourages osteogenesis. A variety of methods were employed to assess the rheological, compositional, and morphological characteristics of the samples: Fourier transform infrared spectroscopy (FTIR) and scanning electron microscopy (SEM) coupled with energy-dispersive X-ray spectroscopy (EDS). Additionally, simulated body fluid (SBF) immersion for bioactivity monitoring and immunocytochemistry for cell viability were used to evaluate the biological response of the scaffolds.

## 1. Introduction

Bone defects, resulting from traumatic injuries, congenital anomalies, or pathological conditions, pose significant challenges in orthopedic and dental practices due to their high prevalence and complexity [1]. The global burden of bone defects is substantial, with millions of individuals affected annually. For instance, the incidence of traumatic bone defects in the United States alone is estimated to be around 6 million cases per year, with a significant portion attributed to sports injuries, vehicular accidents, and falls [2].

The state of the art in bone defect treatment encompasses various strategies, ranging from conservative approaches to advanced regenerative therapies [3]. The primary objective in treating bone defects is to restore the structural integrity and functional capabilities of the affected bone. Traditional treatments include autografts, allografts, and synthetic bone substitutes [4]. Autografts, which involve the transplantation of bone tissue from a donor site to the defect site, are considered the gold standard [5] in bone defect repair due to their osteogenic, osteoconductive, and osteoinductive properties [6]. However, their application is limited by the availability of donor sites, donor site morbidity, and the risk of disease transmission. Allografts, which use bone tissue from a genetically non-identical member of the same species, offer an alternative to autografts but are associated with a higher risk of immune rejection and disease transmission [4].

In recent years, the field of tissue engineering has emerged as a promising avenue for the treatment of bone defects [7]. Scaffolds, typically made from biodegradable polymers, ceramics, or composites, provide a temporary structure that guides bone tissue regeneration [8]. The choice of scaffold material is critical, as it must possess suitable mechanical properties [9], porosity, and bioactivity to support cell attachment, proliferation, and differentiation [10]. The integration of biomaterials, cells, and growth factors in tissue engineering has led to the development of advanced bone graft substitutes that closely mimic the structure and function of natural bone [4]. These bioengineered bone grafts have shown promising results in preclinical and clinical studies, demonstrating their potential to enhance bone regeneration and accelerate the healing process in various bone defect models [11].

Despite the progress made in the field of bone defect treatment, challenges remain, particularly in the areas of personalized medicine [12], the optimization of scaffold design and fabrication techniques [13], and the development of predictive models for patient-specific treatment strategies. Emerging technologies, such as 3D printing [14] and nanotechnology [15], offer new opportunities for the design and fabrication of patient-specific bone graft substitutes that can precisely match the structural and mechanical properties of the native bone tissue [16].

Additive manufacturing, also mainly known as 3D printing [17], is a technique that involves the deposition of successive layers of material on a build bed, resulting in the creation of objects with intricate geometries [18]. In the medical field, tangible models created by additive manufacturing are used to evaluate complex anatomies [19]. Furthermore, this technique can be used to fabricate patient-specific constructs like drill guides, saw guides, and medical implants, which can reduce operating times and enhance the accuracy of surgical procedures [20].

Three-dimensional bioprinting, a subset of additive manufacturing, as defined by Groll et al. [21], is the process of using computer-assisted transfer to pattern and assemble biological materials, including molecules, cells, tissues, and biodegradable biomaterials, combined in a bioink, [22] based on pregenerated perimeters, resulting in the formation of a biofunctional assembly [13]. Klebe demonstrated one of the first models using 3D bioprinting technology in 1988, using a computer-controlled inkjet printer or graphic plotter, described as “cytoscribing” [23], being used to precisely position cells on a 2D substrate [24]. Three-dimensional bioprinting offers numerous advantages considered revolutionary in biomedical engineering. This can automate the layer-by-layer fabrication of cell-laden structures, both in vitro and in vivo [25].

Extrusion-based bioprinting (EBB) has emerged as a popular bioprinting technique, enabling the creation of complex 3D structures with high precision and reproducibility [6]. Fluid dispensing is achieved using mechanical or pneumatic actuation, where the bioink is pushed through a nozzle. The normal force exerted by the downward motion of the plunger induces a rheological response from the bioink, which determines its flow through the nozzle [26]. The flow response of a bioink is vital while developing and screening bioinks [3].

The careful selection of the initial bioink impacts how the construct can be printed, the final cell viability [27], and the mechanical properties of the final device [28]. While the mechanical characterization of printed scaffolds remains crucial for clinical translation, the initial development phase requires comprehensive material characterization to ensure printability and basic biocompatibility [1].

Amongst all contributing factors, rheological properties (shear-thinning, quick shear recovery, adjustable viscosity, pseudoplasticity, thixotropy, substantial yield stress, relatively high viscosity, viscoelastic properties) [29] and printing parameters (printability, printing fidelity, shape retention, long-term shape fidelity, formability) [30] are the primary factors that influence the quality of bioprinted constructs. The printing parameters mentioned above have a great influence on geometric accuracy and cell viability, determining the success of bioprinting [28].

While this study focuses on 3D bioprinting, it is important to acknowledge the emerging field of 4D bioprinting, which incorporates time as the fourth dimension through stimuli-responsive materials that can change shape, properties, or functionality post-printing [9]. The advantages of 4D bioprinting include programmable shape changes, self-assembly capabilities, and dynamic responses to biological environments. However, current limitations include material complexity, limited available stimuli-responsive bioinks, and challenges in controlling temporal responses [9]. Our current approach establishes foundational knowledge that could be extended to 4D applications in future research.

**Figure 1:**
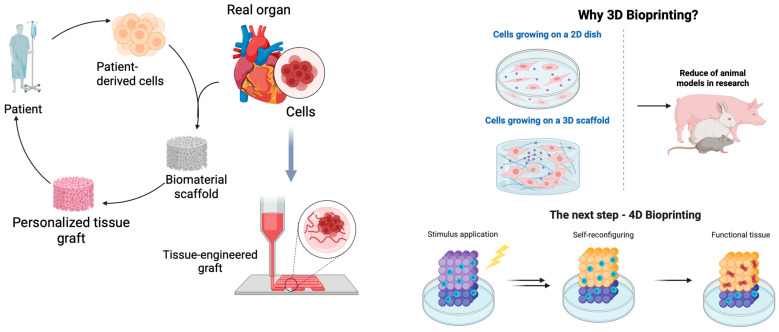
Three-dimensional bioprinting applications and future perspectives (created with Biorender).

Current research focuses on developing biomaterials with favorable biological characteristics, including biocompatibility, non-toxicity, the facilitation of cell migration, proliferation, differentiation, and tissue formation [7]. These properties are crucial for the successful application of bioinks in regenerative medicine and tissue engineering (Figure 1), ensuring safe, effective constructs that support cell growth and differentiation into functional tissues [31].

A class of hydrogels, which are polymeric networks that possess the ability to absorb significant amounts of water or biological fluids [8], with high potential in the biomedical field is based on natural biopolymers [32]. This characteristic arises from the chemical interactions between the polymer chains that constitute the hydrogel, often containing hydrophilic functional groups such as hydroxyl, carboxyl, and amine groups, which attract and bind water molecules through hydrogen bonding and other intermolecular forces [33]. These lightweight mesoporous materials with tunable surface and volume properties offer a unique combination of mechanical strength and 3D surface topography [3].

Regarding the biopolymers used in tissue engineering, alginate is a linear polysaccharide composed of 1,4-β-D-mannuronic acid (M) and α-L-guluronic acid (G) blocks [34]. The M-block segments are flexible, while the G-block segments confer rigidity, with alginate’s viscoelasticity depending on the frequency of these blocks. When exposed to calcium ions (Ca^2+^), alginate forms a three-dimensional network through ionic bonding, known as the “egg box” model [35]. This crosslinking mechanism enhances mechanical strength and stability, making alginate valuable for tissue engineering, drug delivery, and food products [36].

In addition to polysaccharides, gelatin is a water-soluble protein derived from collagen hydrolysis, composed of polypeptide chains with amino acids like glycine, proline, hydroxyproline, and arginine [37]. Its amphoteric nature, due to amine and carboxyl groups, allows for gel formation and interaction with other molecules, creating integrin-binding situses for enhanced cell adhesion [38].

When it comes to oxidic osteogenic materials, the bioresorbable 4S5S bioglass (SiO_2_–P_2_O_5_–CaO–Na_2_O) [39] can be tailored to include various dopants to impart specific properties. The bioglass (BG) doped with silver and europium [40] imparts potent antibacterial properties, as silver ions can disrupt bacterial cell membranes, inhibit DNA replication, and suppress bacterial respiratory chains, leading to the effective control of bacterial growth [41]. This is particularly beneficial in preventing post-surgical infections, a common complication in bone implant procedures [42]. As a result, validating data on the antimicrobial effects of the Eu and Ag-doped bioglass against *S. aureus* and *E. coli* has already been published [40]. Additionally, europium doping is described to induce cell differentiation in mesenchymal stem cells and photoluminescent properties [43]. Upon excitation with ultraviolet light, europium emits visible light, allowing for the use of these bioglass compositions in imaging applications [42]. This feature enables the non-invasive monitoring of bioglass integration with the host bone tissue, providing valuable insights into the progress of bone regeneration [44].

The primary goal of this study is to develop and characterize a novel composite ink comprising an alginate–gelatin hydrogel matrix reinforced with europium and silver-co-doped bioactive glass for extrusion-based 3D bioprinting applications. The novelty lies in the systematic optimization of this specific composite formulation for enhanced printability while maintaining bioactivity and antimicrobial properties. This work addresses the current gap in understanding how co-doped bioactive glass integration affects the rheological and biological properties of natural polymer-based bioinks, providing foundational knowledge for future evaluations.

## 2. Materials and Methods

### 2.1. Materials

Tetraethyl orthosilicate (TEOS, Si(OC_2_H_5_)_4_), triethyl phosphate (TEP, (C_2_H_5_)_3_PO_4_), calcium nitrate tetrahydrate (Ca(NO_3_)_2_·4H_2_O), europium (III) nitrate pentahydrate (Eu (NO_3_)_3_·5H_2_O), sodium alginate, calcium chloride (CaCl_2_), sodium bicarbonate (NaHCO_3_), potassium chloride (KCl), magnesium chloride hexahydrate (MgCl_2_·H_2_O), sodium sulfate (Na_2_SO_4_), chlorohydric acid (HCl), and ethanol were purchased from Sigma-Aldrich (St. Louis, MO, USA). Sodium nitrite (NaNO_2_, purity ≥ 99%) and silver nitrate (AgNO_3_) were procured from Riedel-de Haën (Honeywell, NJ, USA), and nitric acid (HNO₃) was bought from Fluka™ (Honeywell, NJ, USA), while gelatin from porcine skin was bought from Merck (Rahway, NJ, USA).

### 2.2. Hydrogel Synthesis

The chemical composition of the Eu and Ag-doped BG is presented in Table 1, while the sol–gel synthesis route employed and post-processing steps are detailed in a previous work [40].

**Figure 2:**
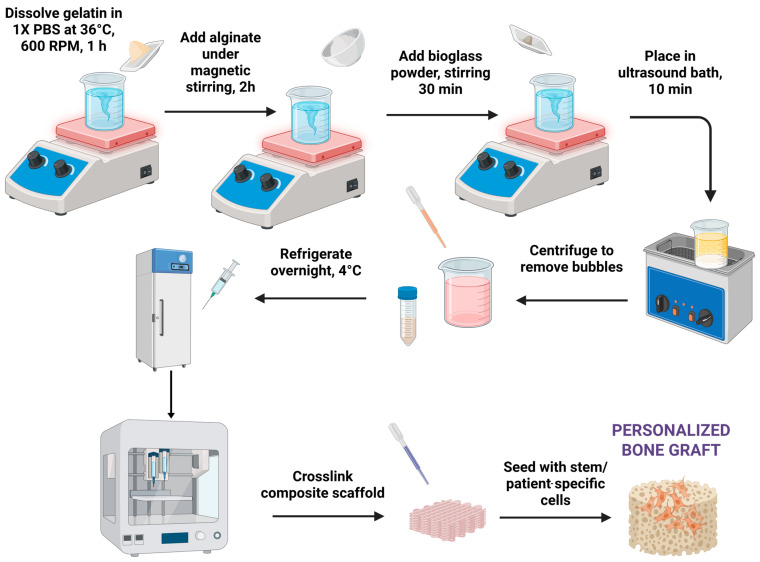
Composite bioglass–hydrogel material synthesis and printing steps.

Regarding the hydrogel matrix, the optimal proportions of alginate and gelatin for 3D printing were determined by testing multiple compositions with varying concentrations of alginate and gelatin. The compositions were tested for printability, viscosity, and shape stability using a Cellink INKREDIBLE+ 3D bioprinter (CELLINK AB, Gothenburg, Sweden), Figure 2. The optimal compositions to be tested consisted of 7% alginate and 8% gelatin and 3% alginate and 7% gelatin, which exhibited good printability, viscosity, and shape stability.

To prepare the hydrogel, PBS 1X was placed in a Berzelius flask with a magnetic stirrer on a heated plate (DLAB MS-H280-Pro, DLAB Scientific Co., Ltd., Beijing, China) at 600 rpm and 36 °C to prevent gelatin denaturation. The desired amount of precursors was added, including sodium alginate (Sigma-Aldrich, CAS-No. 9005-38-3) and gelatin (gelatin from porcine skin, CAS-No. 9000-70-8, Sigma-Aldrich). Once the gelatin was completely dissolved, alginate was added, and the mixture was stirred for an additional 2–3 h to ensure complete incorporation.

The inorganic phase entailed the introduction of small amounts of Eu-doped bioglass powder, as seen in Table 2. Prior to addition, the bioglass powder was passed through a standard laboratory sieve (mesh size 0.45 μm) to ensure a uniform particulate size. The sieved powder was then added to the organic solution under continuous magnetic stirring. To achieve the thorough dispersion of the bioglass particles within the hydrogel matrix, the mixture was subjected to ultrasonication. An ultrasonic bath (Elmasonic S30H, Elma Schmidbauer GmbH, Singen, Germany) was employed, where the solution underwent three cycles of ultrasound treatment, each lasting for 10 min.

Upon the completion of the ultrasonication process, the homogenized hydrogel precursor solution was transferred to the barrel of a bioprinter syringe. To remove entrapped air bubbles and ensure homogenous hydrogel density, the syringe was manually centrifuged using a 3D-printed manual centrifuge. The resulting degassed hydrogel was then stored at 4 °C to facilitate gelation. The gelation process was allowed to proceed overnight to yield a fully structured hydrogel suitable for subsequent bioprinting applications.

### 2.3. Three-Dimensional Printing Process

The biomaterials were printed using an extrusion-based 3D bioprinter from, through a 22 G conical nozzle, and 10 × 10 mm^2^ square grid-shaped scaffolds were obtained in 90 mm Petri dishes. The 10 × 10 mm^2^ dimensions were selected based on standard in vitro testing protocols for bone scaffold evaluation and to ensure adequate surface area for cell seeding while maintaining material efficiency. The 4-layer configuration was chosen to achieve a target thickness of approximately 2 mm, which provides sufficient structural integrity while allowing for nutrient diffusion throughout the scaffold thickness, as recommended for bone tissue engineering applications.

**Figure 3:**
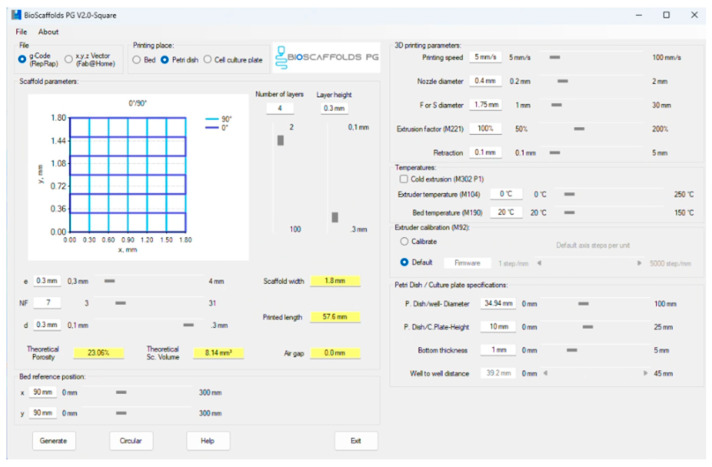
Three-dimensional printing parameter settings and GCode generation through Bioscaffolds V2 interface.

In order to generate GCode information, Bioscaffolds V2.0 software (Figure 3) was used. The optimal printing parameters for the obtained compositions are described in Table 2. Scaffold porosity was controlled through printing parameters including layer height (0.5 mm), infill density (60%), and strand spacing (0.8 mm). The grid pattern inherently creates interconnected pores, with additional microporosity arising from the hydrogel matrix structure.

### 2.4. Characterization Methods

#### 2.4.1. Rheological Evaluation

The rheological properties of the samples were analyzed to evaluate their suitability for bioprinting applications in an extrusion-based system.

Each sample was pre-conditioned for 2 min at the testing temperature before measurement initiation. Three replicates were performed for each composition, with a 30 s equilibration time between each measurement point.

Rheological measurements were obtained using an Anton Paar rheometer with a 25 mm diameter parallel-plate geometry. Hydrogel samples, consisting of 6% alginate and 3% gelatin, were loaded onto the bottom plate of the rheometer with a 0.5 mm gap. In addition, tests were also conducted on the composite samples, with negligible differences. The tests were performed at a controlled temperature of 25 °C ± 0.1 °C using a temperature-controlled Peltier system. The shear rate was varied from 0.1 to 100 s^−1^ following a logarithmic progression with point durations from 3 to 20 s. Measurements were conducted according to the ASTM D4440 standard [45] protocol for the rheological properties of non-Newtonian materials.

Viscosity and shear stress as a function of shear rate were recorded, with the shear rate set to 1–50 s^−1^. Shear stress vs. shear rate graphs were used to determine the dynamic yield stress by fitting the Herschel–Buckley model to the data with the following equation:

(1) $$\sigma \;=\;K_{\gamma^{n}}\;+\;\sigma_{o}$$

where σ is shear stress, *γ* is the shear rate, *K* is the consistency coefficient, *n* is the flow behavior index, and σ_*o*_ is the dynamic yield stress.

#### 2.4.2. Scanning Electron Microscopy and Energy-Dispersive X-ray Spectroscopy

Scanning electron microscopy (SEM) is viewed as a non-invasive method for examining the morphological characteristics of materials. These examinations were conducted using the FEI Inspect F50 SEM equipped with an energy-dispersive X-ray spectrometer (EDS), which enables the analysis of the elemental composition of the samples. Given that each element has a unique energy spectrum, this technique allows for the identification of the constituent elements. Additionally, the intensity of the spectral peaks provides insights into the relative abundance of these elements within the sample.

Prior to analysis, the samples were prepared by coating them with a thin layer of gold to enhance their conductivity, applied through a process called DC magnetron sputtering.

#### 2.4.3. Fourier Transform Infrared Spectroscopy

Fourier transform infrared spectroscopy (FTIR) is a technique commonly used for the physicochemical characterization of organic materials. Each absorption band corresponds to a mode of vibration of a chemical bond between two atoms, and if a chemical bond is characteristic of a constituent, it can be considered a tracer of that constituent.

Measurements were conducted separately on the bioglass dried gel and synthesized powder, using the FTIR Jasco spectrometer, at room temperature using the attenuated total reflection (ATR) module, with 32 scans of the samples between 4000 and 500 cm^−1^ at a resolution of 4 cm^−1^. Samples were dried and then placed on the attenuated total reflection accessory of the spectrometer. In addition, FTIR spectra were attained for the bioglass powder in previous experiments, and the results of FTIR are presented in our previous work [40].

#### 2.4.4. Optical Microscopy—Printing Accuracy

**Figure 4:**
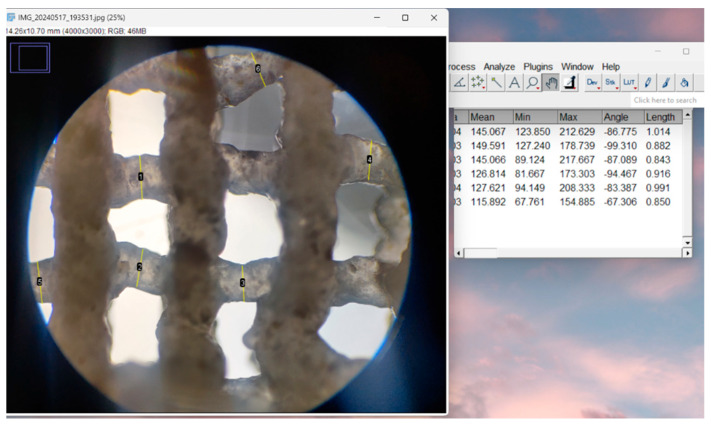
Printability evaluation using strand measurement through ImageJ software.

The assessment of the printing accuracy for the 3D-printed scaffolds was carried out using optical microscopy, as seen in Figure 4. Furthermore, to quantitatively assess the precision of the printing process, the strand thickness of the gel within the scaffolds was analyzed by comparing the measured strand dimensions with the nozzle diameter. Utilizing ImageJ (Java 8) software, the strand thickness was measured with its built-in Analysis function.

#### 2.4.5. Gravimetric Method

The swelling behavior of the scaffolds was determined using the gravimetric method. The scaffolds were initially weighed in a dry state and then submerged in 1X PBS solution. After a 4 h period, ensuring the complete absorption of PBS, the scaffolds were weighed again. The swelling capacity of the gel was quantified using the following equation:

(2) $$SD\%=\frac{W_{wet}-W_{dry}}{W_{dry}}\times 100,$$

where W_wet_ represents the weight of the scaffold after immersion in PBS, and W_dry_ represents the weight of the scaffold before being submerged [10].

#### 2.4.6. In Vitro Degradation Study

To evaluate the degradation profile, both pristine scaffolds (gelatin and alginate) and composite scaffolds incorporating bioglass were immersed in 3 mL of PBS 1X at 37.1 °C. The scaffolds were weighed at regular intervals, on the first, third, seventh, fourteenth, and twenty-eighth days, to monitor the degradation process over time.

#### 2.4.7. Porosity Evaluation with Particle Analyzer

**Figure 5:**
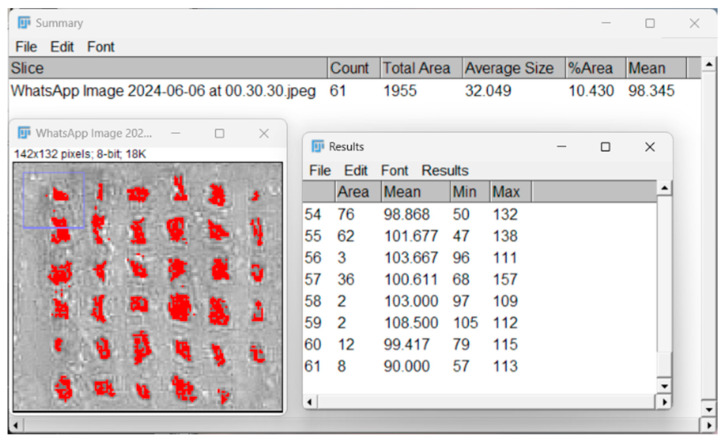
Porosity evaluation through ImageJ software.

The porosity of the 3D-printed scaffolds was evaluated using ImageJ software, as illustrated in Figure 5. First, the digital images of the scaffolds were subjected to the Threshold function, which allowed for the segmentation of the scaffold solid and void regions by setting a specific contrast range. This step enabled the distinction between the polymer matrix and the pore spaces, converting the grayscale image into a binary format.

Subsequently, the Particle Analyzer tool was employed to quantify the porosity parameters. This feature of ImageJ analyzed the binary image, counting and measuring the individual pore areas, as well as their distribution within the scaffold.

#### 2.4.8. In Vitro Mineralization

The methodology for assessing the biological activity of doped bioglass thin films involved several key procedures. Initially, a simulated body fluid (SBF) was prepared using the method outlined by Kokubo et al. [46]. Following this, the sample, weighing 30 mg, was carefully measured and placed into a sterile vessel. The sample was then completely submerged by adding 1.5 mL of the SBF solution that had been prepared. The submerged sample was then subjected to an incubation period at 37 °C for a duration of 28 days. Upon the completion of this immersion phase, the sample was carefully removed from the SBF solution and meticulously rinsed with deionized water. Finally, the sample underwent analysis using scanning electron microscopy (SEM) to evaluate its biological response.

To assess in vitro mineralization and apatite formation, the composite scaffolds were placed in a 12-well plate and submerged in 3 mL of 1.5X SBF, prepared according to the protocol outlined by Kokubo et al. [46] (Table 3). The samples were then incubated in a heated incubator at 37.1 °C. After a duration of 14 days, the samples were examined under an optical microscope for analysis.

#### 2.4.9. Cell Seeding and LIVE/DEAD Assay

Human fetal osteoblastic cells (hFOB 1.19) were cultured in a humidified chamber (95% air; 5% CO_2_) at 37 °C in Dulbecco’s Modified Eagle’s Medium (DMEM) and supplemented with 10% fetal calf serum and 1% penicillin–streptomycin. Once in confluence, cells were trypsinized and counted. The scaffolds were sterilized via UV-C for 20 min on each side and immersed in cell medium. On top of each scaffold, 2 × 10^5^ cells were seeded with 2000 μL of DMEM in each well and were incubated at 37 °C for 48 h. For the LIVE/DEAD cell viability test, the manufacturer (Invitrogen) protocol was followed [47].

The growth medium was removed from the scaffold samples, and they were gently rinsed with phosphate-buffered saline (PBS) to eliminate any detached cells or debris. A fresh staining solution was prepared by combining equal volumes of the diluted Calcein AM and Propidium Iodine solutions. The LIVE/DEAD staining solution was added to each sample, and they were incubated at room temperature in the dark for 30–60 min to facilitate the penetration of the dyes into the cells and their binding to the respective intracellular targets [47].

Following incubation, the samples were rinsed with PBS to remove any unbound stain. The samples were examined using a fluorescence microscope (Zeiss LSM) equipped with filters suitable for detecting green and red fluorescence. Image analysis software, ZEN Studio 1.6.1, was utilized to quantify the number of live (green) and dead (red) cells. The fluorescence intensity was measured, or the cells in each color channel were manually counted, and the viability percentage was calculated using the following formula:

*Viability Percentage* = (*Number of Live Cells*/*Total Number of Cells*) × 100. (3)

## 3. Results

### 3.1. Three-Dimensional Printing Process

**Figure 6:**
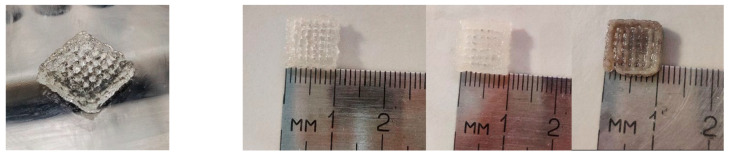
Morphology, shape, and measurements of 3D-printed scaffolds P2-P5.

The 10 × 10 mm^2^, 4-layered porous scaffolds were obtained using EBB 3D printing technology. Figure 6 displays their shape stability, with their design integrity being maintained over time, at room temperature. The printability of these scaffolds was demonstrated by the lack of gaps or air bubbles in the filament strands.

The composite structure of the scaffolds was achieved, and the porosity was controlled to facilitate cell adhesion, proliferation, and the penetration of cells and the extracellular matrix essential for tissue regeneration.

### 3.2. Characterization Methods

#### 3.2.1. Rheological Evaluation

**Figure 7:**
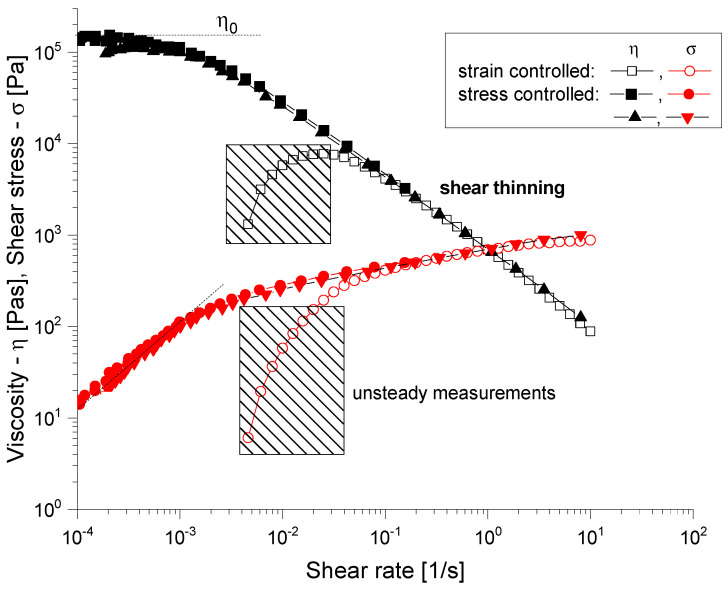
Rheological characterization of uncrosslinked hydrogels. Viscosity as function of shear rate, from 1 to 50 s^−1^ (black). Shear stress as function of shear rate, from 1 to 50 s^−1^ (red).

Viscosity and shear stress were plotted against shear rate in log–log, as illustrated in Figure 7, revealing a pronounced shear-thinning behavior, as evidenced by the rapid decrease in viscosity with an increasing shear rate. In the context of extrusion-based bioprinting, the material’s viscosity and its shear-thinning properties will determine how easily it will flow through the printer’s nozzle. The observed shear-thinning behavior (Figure 7) demonstrates a power-law relationship with flow index values ranging from 0.3 to 0.4, indicating moderate pseudoplastic behavior compared to typical alginate-based bioinks (n = 0.2–0.6). The viscosity values at low shear rates (10–50 Pa·s) fall within the optimal range for extrusion-based bioprinting, being significantly higher than those for pure alginate solutions (1–5 Pa·s) but lower than those for high-concentration gelatin gels (100–500 Pa·s). This intermediate viscosity profile ensures adequate printability while maintaining structural integrity during deposition.

#### 3.2.2. Scanning Electron Microscopy and Energy-Dispersive X-ray Spectroscopy

**Figure 8:**
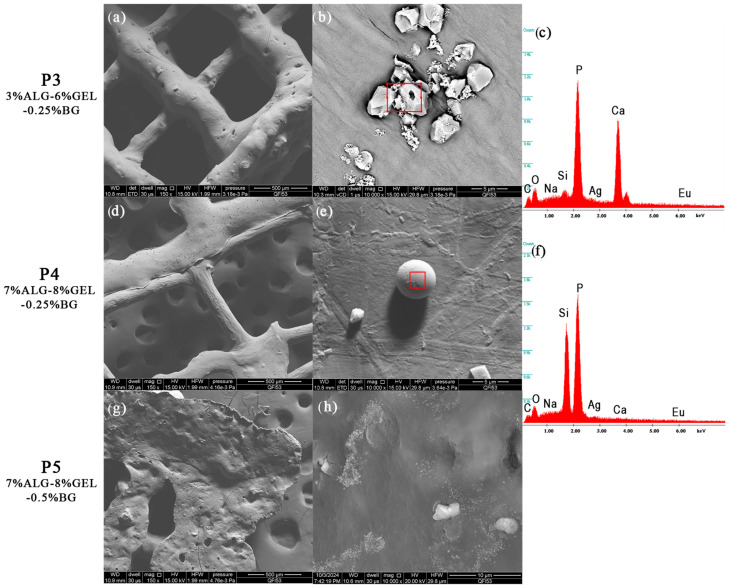
Scanning electron microscopy images of the 3D-printed composite scaffolds: (**a**,**b**,**d**,**e**,**g**,**h**). EDS spectra: (**c**,**f**).

The scanning electron microscopy (SEM) images in Figure 8 provide a comprehensive view of the microstructural characteristics of the composite scaffolds. The P3 sample, composed of 3%Alg-6%Gel-0.25%BG, exhibits a well-defined, porous, interconnected structure with a relatively smooth surface texture punctuated by small nodular features. The bioglass particles appear to be successfully integrated into the polymer matrix during the fabrication process.

When examining the P4 formulation (7%Alg-8%Gel-0.25%BG), the increased polymer concentration manifests in a more pronounced porous network with added bioglass agglomerates well-embedded within the polymeric structure. This enhanced structural definition can be attributed to the higher concentration of both alginate and gelatin components, which contribute to a more robust scaffold architecture.

The P5 sample (7%Alg-8%Gel-0.5%BG), containing the highest bioglass concentration, demonstrates notably different surface characteristics. The increased bioglass content results in a rougher surface topology with more pronounced surface irregularities. The pore structure appears less uniform compared to P3 and P4, and there is evidence of increased particle aggregation, likely due to the higher concentration of bioglass particles in the composite. This increase in porosity and surface roughness is a direct result of the incorporation of bioglass particles into the hydrogel matrices. The primary disadvantages of bioglass include mechanical weakness and low fracture resistance due to its amorphous two-dimensional glass network structure. The bending strength of most bioglass ranges from 40 to 60 MPa, which is insufficient for load-bearing applications. Additionally, bioactive glass scaffolds remain brittle and therefore unsuitable for grafting applications in sites subjected to cyclic loads. However, these mechanical limitations can be mitigated through composite formulations with polymeric matrices, as demonstrated in our study, or by restricting applications to non-load-bearing sites.

The EDS analysis provides valuable insights into the elemental composition of the scaffolds. In sample P3, strong peaks for phosphorus (P) and calcium (Ca) indicate the presence of calcium phosphate phases, while the silicon (Si) peak confirms the successful incorporation of bioglass. The presence of sodium (Na) and oxygen (O) can be attributed to the alginate structure, and trace elements such as silver (Ag) and europium (Eu) are also detected. The P4 sample shows similar elemental constituents but with notably different peak intensities, particularly showing dominant phosphorus and silicon signals with reduced calcium intensity compared to P3. This variation in elemental distribution suggests that the different polymer and bioglass concentrations influence not only the physical structure but also the chemical composition of the resulting scaffolds. Finally, the presence of gold is a result of the deposited layer intended to ensure the sample’s conductivity [40].

A comparative analysis of the SEM and EDS results reveals significant changes in both the structural and compositional properties of the scaffolds upon the introduction of bioglass. The increased porosity and surface roughness observed in the SEM images of the composite scaffolds are likely to enhance their mechanical properties and surface area, which are crucial factors for cell adhesion, proliferation, and tissue regeneration. Furthermore, the presence of bioglass, as evidenced by the EDS data, introduces bioactive elements such as silicon and calcium that can promote osteogenic activity and improve the overall biological performance of the scaffolds.

#### 3.2.3. Fourier Transform Infrared Spectroscopy

**Figure 9:**
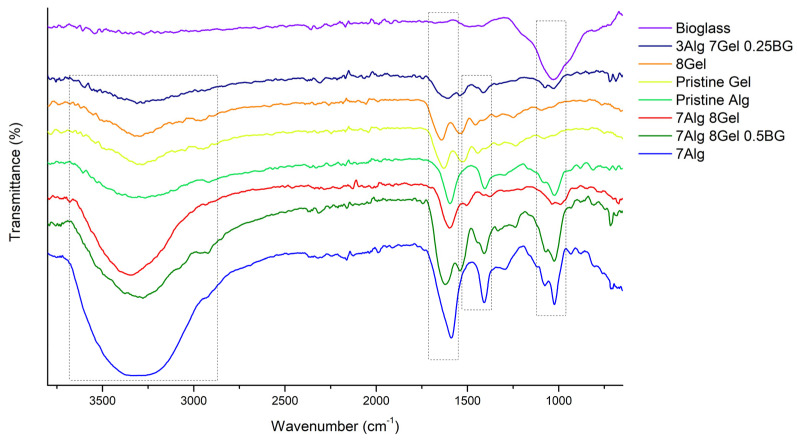
FTIR spectra of hydrogel compositions.

The FTIR spectrum of raw alginate (Figure 9) typically exhibits several characteristic peaks. The broad absorption band around 3200–3400 cm^−1^ corresponds to O-H stretching vibrations, indicative of hydroxyl groups. The peak near 1410 cm^−1^ corresponds to the symmetric stretching of carboxylate groups. Additionally, C-O stretching vibrations appear near 1030 cm^−1^. Gelatin shows distinctive FTIR peaks corresponding to its proteinaceous nature. The amide II band, arising from N-H bending and C-N stretching vibrations, is observed around 1526 cm^−1^, while the band around 3200–3400 cm^−1^ corresponds to O-H.

In the FTIR spectrum of the varied alginate–gelatin–bioglass scaffolds, the characteristic peaks of each component can be observed in Table 4, confirming their successful incorporation. The broad O-H stretching band (3200–3400 cm^−1^) and the carboxylate stretching bands (1600 and 1410 cm^−1^) from alginate are present. The amide bands (1650 cm^−1^ for amide I, 1550 cm^−1^ for amide II, and 1440 cm^−1^ for amide III) from gelatin are also clearly visible. Additionally, the Si-O-Si stretching bands from bioglass (around 1025 cm^−1^) are evident in the scaffold spectrum, indicating the presence of bioglass within the polymer matrix [40]. The overlapping of bands and slight shifts in peak positions suggest interactions between the components, likely through hydrogen bonding and electrostatic interactions.

Comparative analysis reveals distinct spectral differences between doped and un-doped bioactive glass compositions. The europium-doped samples show additional absorption at 1048 cm^−1^, attributed to the Si-O-Si peak, also shown at 1032 to 1028 cm^−1^, indicating structural modifications in the silicate network. These spectral changes are consistent with successful dopant incorporation and align with previous studies on rare earth-doped bioactive glasses.

#### 3.2.4. Optical Microscopy—Printing Accuracy

**Figure 10:**
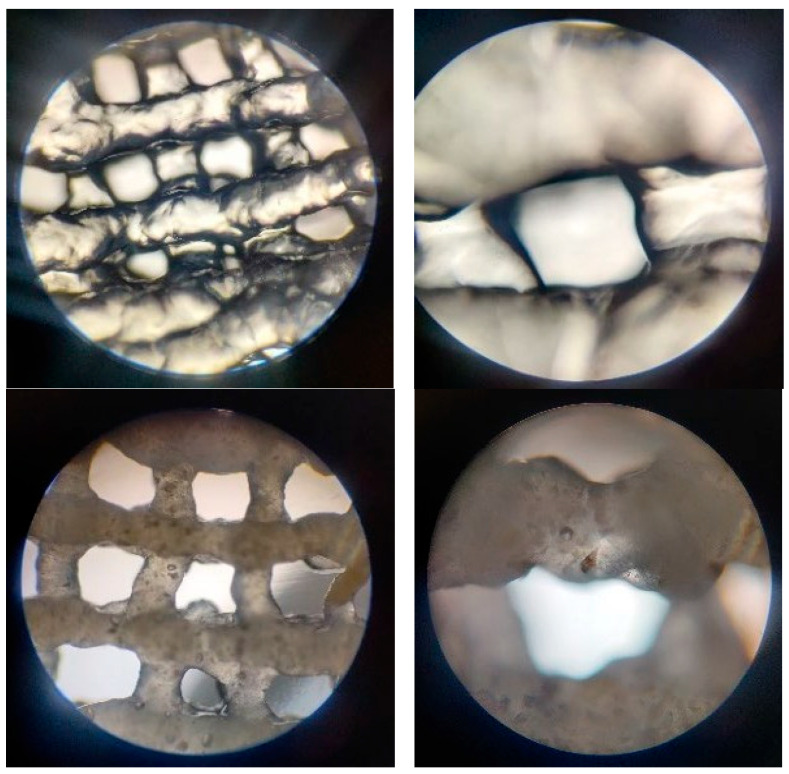

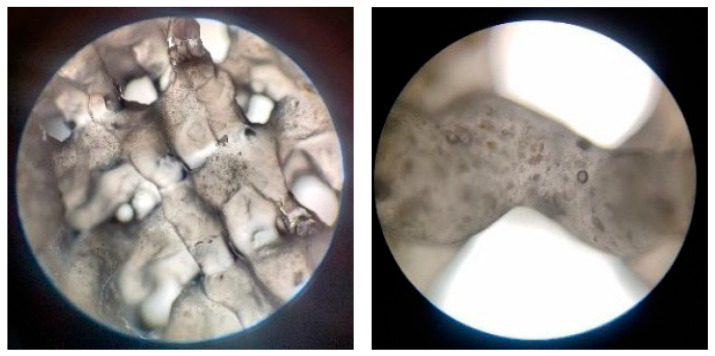
Optical microscopy images of the 3D-printed scaffolds: P2, magnification 4×, 10×; P3, magnification 4×, 10×; P4 magnification 4×, 10×.

In Figure 10 at 4× magnification, the optical microscopy images revealed a homogeneous dispersion of bioglass particles throughout the polymeric matrix. The particles exhibited diverse shapes and sizes. The polymeric alginate and gelatin scaffolds displayed a smooth, continuous structure, indicating a uniform printing process. Higher-magnification images (10×) further elucidated the morphology of the scaffolds. The bioglass particles were observed to be well-integrated within the polymeric matrix, with no evidence of agglomeration or clustering. The particles’ surface appeared irregular, with some displaying angular geometries. The polymeric matrix surrounding the bioglass particles exhibited a uniform structure, suggesting a high degree of interconnectivity. The homogeneous distribution of bioglass particles and the smooth texture are crucial for ensuring consistent bioactivity and mechanical reinforcement across the scaffold. The scaffolds’ pore structure displayed a decent level of fidelity to the original 3D model, indicating a successful printing process.

**Figure 11:**
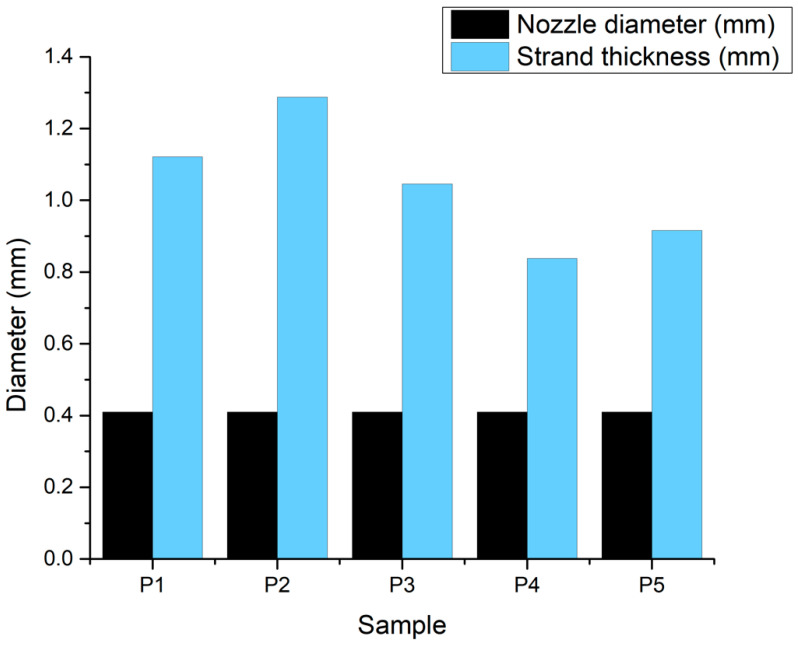
Print accuracy chart.

In addition, the values obtained for the strand thickness, as displayed in the chart in Figure 11, suggest that the hydrogel expanded by approximately double the diameter of the 22 G nozzle. This could be explained by the non-Newtonian nature of the complex fluids involved, which would cause the shear-thinning effect to manifest. However, this phenomenon seems to be slightly reduced in composite materials.

#### 3.2.5. Swelling Degree

**Figure 12:**
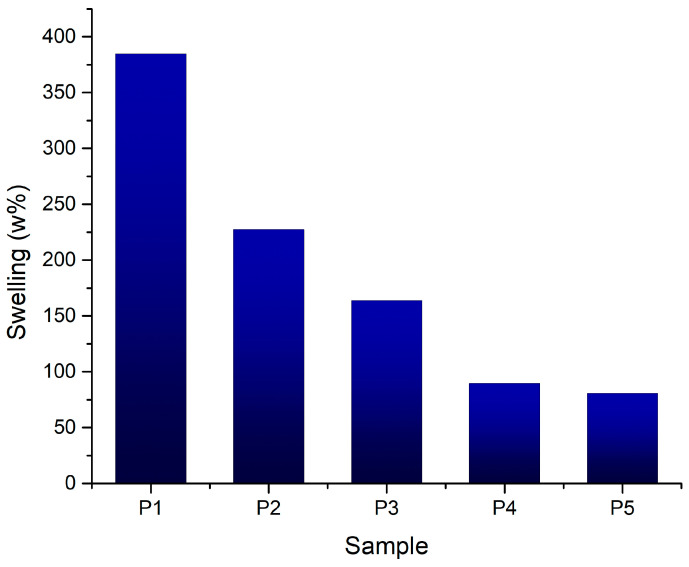
Swelling values for the 3D-printed samples.

The swelling degree of polymeric scaffolds is a critical parameter influencing their performance in tissue engineering applications. The results (Figure 12) indicate that the incorporation of bioglass particles into alginate and gelatin scaffolds modulates the swelling behavior, with higher bioglass content leading to reduced swelling. This trend suggests that bioglass particles restrict the polymer network’s ability to absorb water, thereby reducing the overall swelling capacity. Across all samples, the swelling degree remained below 250%, indicating a moderate water absorption capacity. The controlled swelling behavior of these scaffolds is critical for tissue engineering applications, as excessive swelling can lead to scaffold degradation and compromised mechanical properties.

#### 3.2.6. Degradation Rate

**Figure 13:**
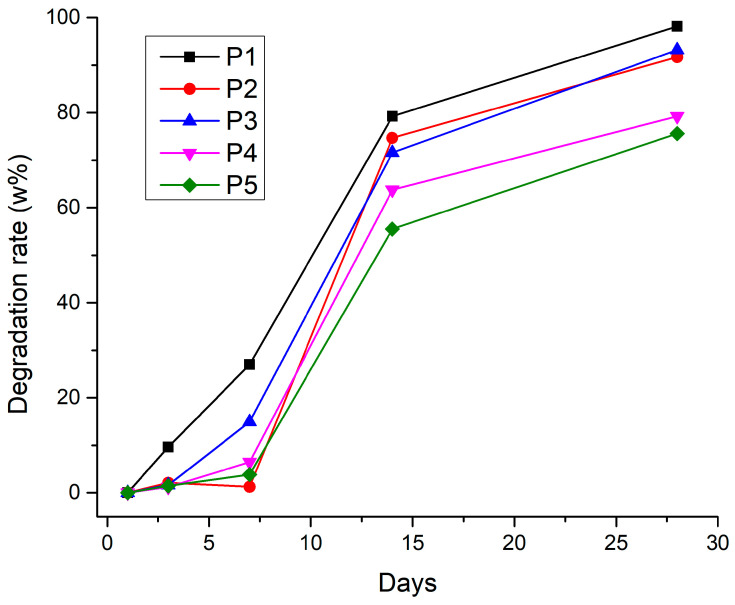
Degradation rate for 3D-printed samples.

The degradation test results highlight the significant breakdown of all tested materials over 28 days (Figure 13). The gelatin scaffold degrades more rapidly, suggesting that gelatin, on its own, has a faster degradation rate under the test conditions. This could be due to the absence of other components that might slow down this process (such as alginate’s resistance to enzymatic degradation or bioglass’s ability to modulate the local pH). This correlates with the fact that the gelatin sample also has the highest value of porosity.

#### 3.2.7. Porosity Evaluation

**Figure 14:**
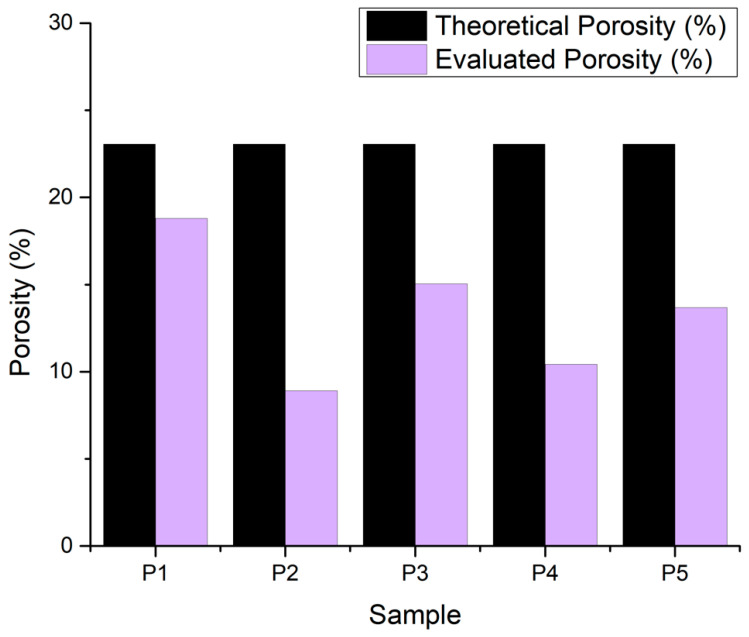
Porosity for the 3D-printed samples.

The theoretical value of porosity is suggested by BioScaffolds V2 as 23.07% for the 22 G nozzle. The resulting values are lower than anticipated due to the expansion of the filament strand after being extruded. A porosity lower than 25% across all samples (Figure 14) suggests a set of materials that are relatively dense and compact, which can be advantageous for certain applications requiring high strength, durability, or resistance, such as cortical bone areas. For bone tissue engineering, multi-layered scaffolds with smaller pores (50–100 μm) foster cell attachment, while larger pores (200–400 μm) enhance nutrient diffusion and angiogenesis. Moderately porous scaffolds (60–80% porosity) are optimal for cancellous bone regeneration, supporting uniform osteogenesis throughout the construct. High-porosity scaffolds (>80%) facilitate rapid vascularization but may compromise mechanical integrity. The specific application determines optimal porosity: cortical bone defects benefit from lower porosity (20–40%) for load-bearing capacity, while trabecular bone regeneration requires higher porosity (60–80%) for enhanced cellular infiltration and nutrient transport.

#### 3.2.8. In Vitro Mineralization

**Figure 15:**
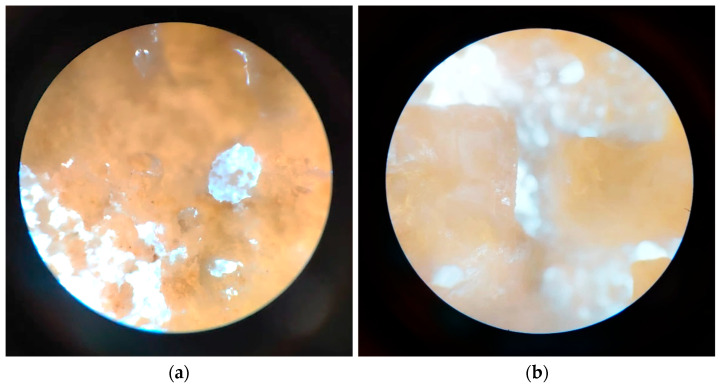
Optical microscopy images of the 3D-printed scaffolds: (**a**) P4, magnification 10×; (**b**) P5, magnification 10×; after 14 days in SBF 1.5X.

Regarding the composite printed samples (Figure 15), after 14 days in SBF 1.5X (1.5 times the concentration of phosphate ions compared to normal SBF), apatite formations are present on the composite scaffolds, which is to be expected, as the bioactive glass shows the behavior of mineralization, which results in good osteoinductive properties for the scaffolds.

#### 3.2.9. Cell Seeding and LIVE/DEAD Assay

**Figure 16:**
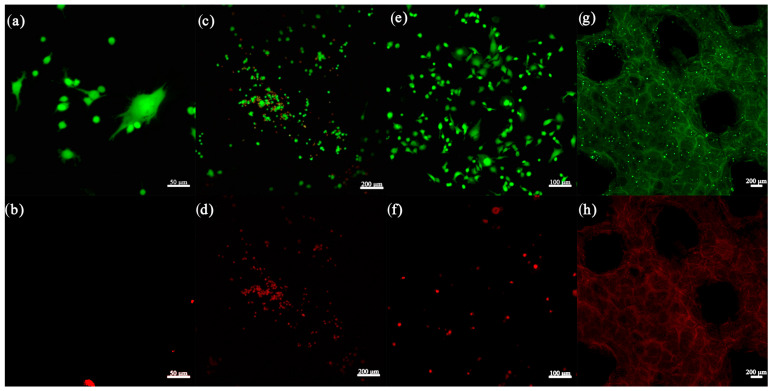
Confocal microscopy images of the 3D-printed scaffolds: (**a**,**b**) control, (**c**,**d**,**e**,**f**) 8% Gel—7% Alg scaffold, and (**g**,**h**) 6% Gel—3% Alg—0.25% BG.

The samples which demonstrated the best stability for manipulation, after 48 h incubation, were further evaluated for cell viability. Figure 16 illustrates good cell viability based on the green color of the fluorescence from Calcein AM, for both the hydrogel and composite scaffolds. In addition, osteoblast adhesion was observed and attributed to cell-binding moieties from gelatin and ion release from the bioglass composition, and cell migration was visible from the difference in focus in the Z-stack. The distribution of viable cells shows promising results for the composite scaffolds, but further in vitro evaluations, such as MTT assays and immunocytochemistry, are required to assess their biological potential.

## 4. Discussion

Our findings align with previous research demonstrating the enhanced bioactivity of europium and silver-co-doped bioactive glasses. Chanuka et al. reported that 45S5 bioactive glass scaffolds loaded with bioactive compounds showed superior bone regeneration compared to control groups, with increased bone mineral density after 8 weeks [48]. Similarly, Feng et al. demonstrated that bioactive glass-functionalized hydrogels promoted vascular network formation and enhanced osteoblast proliferation [49]. The antimicrobial properties of silver-doped bioactive glass have been extensively validated, with studies showing effective control against *S. aureus* and *E. coli* while maintaining biocompatibility.

Our rheological characterization results are consistent with Karvinen et al., who reported optimal viscosity ranges of 10–50 Pa·s for extrusion-based bioprinting applications [50]. The observed shear-thinning behavior with flow index values of 0.3–0.4 falls within the range reported for successful bioprinting formulations. The combination of natural polymers with bioactive ceramics was previously explored by Kaliampakou et al., who emphasized the importance of matching degradation rates between components for optimal tissue integration [51].

While our preliminary cell viability results demonstrate promising biocompatibility, the complete biological characterization of these composite scaffolds requires comprehensive in vitro evaluation protocols. Future studies will systematically assess metabolic activity, proliferation kinetics, and osteogenic differentiation markers to establish the full therapeutic potential of the europium and silver-co-doped bioactive glass composite scaffolds.

## 5. Conclusions

As the research in this field continues, there is a need to optimize the printability of composite hydrogels with doped bioglass (7% alginate, 8% gelatin, 0.25% BG—Alg-Gel-BG) by tuning parameters such as the bioink composition, printing parameters, and post-processing conditions. Additionally, further studies are required to evaluate the effects of scaffold architecture on long-term performance and biocompatibility in vitro. By addressing these aspects, personalized 3D bioprinting has the potential to provide effective solutions for bone defect regeneration.

Cell adhesion is the main requirement in a number of cellular processes involved in tissue repair, such as cell diffusion, migration, proliferation, and differentiation. Designing applications for bone tissue regeneration requires an understanding of the variables that influence cell behavior as well as how to manage it, including adhesion, orientation, migration, and differentiation on scaffolds.

In terms of prospects, these strategies hold tremendous potential for tissue engineering applications to enhance stem cell differentiation, to locally program cell proliferation and apoptosis to drive morphological development, or to trigger growth factor receptor signaling that mediates structural self-organization. Overall, both the understanding of how the cellular mechanical environment affects cell and tissue behavior and the technical advances required to reproduce these mechanical microenvironments in vitro are advancing rapidly. However, most of these technical advances are still in their infancy, and making them widely accessible and applicable in a high-throughput manner will still face many challenges.

Current technological challenges in bioactive scaffold development include limited printable biomaterial options, a lack of standardized bioprinting protocols, and scale-up difficulties for clinical translation, due to tissue complexity. Key limitations include printing resolution constraints that prevent the replication of native tissue microarchitecture, particularly for vascular networks requiring sub-10 μm features.

However, some of these challenges can be initially addressed through the development of standardized bioink libraries with quality control systems to ensure reproducibility and safety; the integration of growth factor delivery systems and angiogenic strategies to promote vascularization; and the development of automated, scalable manufacturing processes that maintain biological functionality while reducing production costs.

In addition, cells can be engineered to express genes with synthetic ion-sensitive promoters, which allows for a further regulation of cellular responses induced by channel modulation. Although not yet practically implemented for tissue engineering applications, it seems feasible that scaling up such approaches could precisely control or even synthetically regulate mechanosensitive cellular responses within in vitro or implanted tissue constructs.

Future studies should address several aspects that may interfere with the veracity of the results obtained. First, the precise correlation between the types of mechanical stresses and their signaling pathways can be further explored by conditions simulating mechanical stresses in vitro using 3D cultures and bone biomimetic materials.

## Figures and Tables

**Table 1:** Bioglass composition expressed in molar percentages.

Bioglass Composition (mol%)					
65.0	4.5	2.5	24.0	1.0	3.0
SiO_2_	P_2_O_5_	Na_2_O	CaO	Ag_2_O	Eu_2_O_3_

**Table 2:** Hydrogel composition and 3D printing parameters.

Sample	Hydrogel Composition			pH	Pressure (kPa)	Nozzle Diameter	Printing Speed	Layers	Crosslinking Time
	Alginate	Gelatin	Eu-Doped BG						
P1	3%	7%		8	25	22 G	20 mm/s	4	5 min
P2	7%	8%		7	135				
P3	3%	6%	0.25%	8	170				
P4	7%	8%	0.50%	8	210				
P5	7%	8%	0.25%	8	225				

**Table 3:** Composition of SBF 1.5X.

Order	Reagent	Quantities for 100 mL SBF 1.5X
#0	Ultra-pure water	75 mL
#1	NaCl	1.1994 g
#2	NaHCO_3_	0.0525 g
#3	KCl	0.0336 g
#4	K_2_HPO_4_·3H_2_O	0.0342 g
#5	MgCl_2_·6H_2_O	0.0458 g
#6	1 kmol/m^3^ HCl	6 cm3
#7	CaCl_2_	0.0417 g
#8	Na_2_SO_4_	0.0107 g
#9	(CH_2_OH)_3_CNH_2_	0.9086 g
#10	1 kmol/m^3^ HCl	Appropriate amount for adjusting pH

**Table 4:** Correlated bonds of FTIR spectra.

Alg	Gel	BG	Bands (cm^−1^)	Correlated Bonds
	8%		1631.48	Amide II
			1545.67	Amide I
			1414.53	(CO_3_)^−2^
7%			3325.64	O-H
			1594.84	O-C-O
			1414.53	(CO_3_)^−2^
7%	8%		3325.64	O-H
			1631.48	Amide II
			1545.67	Amide I
			1442.49	C-H
3%	7%	0.25%	1594.84	O-C-O
			1414.53	(CO_3_)^−2^
			1078.98	Si-O-Si
			1031.73	Si-O-Si
7%	8%	0.50%	3299.61	O-H
			1629.55	Amide II
			1526.38	Amide I
			1414.53	(CO_3_)^−2^
			1078.98	Si-O-Si
			1031.73	Si-O-Si

## Data Availability

The raw data supporting the conclusions of this article will be made available by the authors on request.

## Abbreviations

The following abbreviations are used in this manuscript:

FTIR	Fourier transform infrared spectroscopy
SEM	scanning electron microscopy (SEM)
EDS	energy-dispersive X-ray spectroscopy
SBF	simulated body fluid
EBB	extrusion-based bioprinting
Alg	alginate
Gel	gelatin
BG	bioglass
DNA	deoxyribonucleic acid
hFOB 1.19	human fetal osteoblastic cells
TEOS	tetraethyl orthosilicate Si(OC_2_H_5_)_4_
TEP	triethyl phosphate (C_2_H_5_)_3_PO_4_
DC	direct current
ATR	attenuated total reflection
PBS	phosphate-buffered saline
DMEM	Dulbecco’s modified Eagle’s medium
MTT	3-(4,5-dimethylthazolk-2-yl)-2,5-diphenyl tetrazolium bromide
